# Sudden gains in face-to-face and internet-based cognitive therapy for social anxiety disorder

**DOI:** 10.1016/j.brat.2023.104334

**Published:** 2023-07

**Authors:** Graham R. Thew, Anke Ehlers, David M. Clark

**Affiliations:** a Department of Experimental Psychology, University of Oxford, UK; b Oxford University Hospitals NHS Foundation Trust, Oxford, UK; c Oxford Health NHS Foundation Trust, Oxford, UK

**Keywords:** Sudden gains, Social anxiety, Internet, Cognitive behaviour therapy, Attention

## Abstract

Sudden gains are large and stable decreases in clinical symptoms between consecutive therapy sessions. This work examined the frequency and possible determinants of sudden gains in Cognitive Therapy for Social Anxiety Disorder, comparing face-to-face (CT) and internet-based (iCT) formats of treatment delivery. Data from 99 participants from a randomised controlled trial were analysed. The frequency of sudden gains was high: 64% and 51% of participants experienced a sudden gain in CT and iCT respectively. Having a sudden gain was associated with lower social anxiety symptoms at posttreatment and follow-up. There was evidence of reductions in negative social cognitions and self-focused attention immediately prior to the sudden gain, contrasting with no prior reductions in depression symptoms. Ratings of session videotapes in CT showed that clients' statements indicated greater generalised learning in sessions immediately prior to gains, compared to control sessions. This may suggest a role for generalised learning in facilitating these large symptom reductions. There were no significant differences in results between the CT and iCT treatment formats, suggesting that the therapy content appears to play a more important role in determining participants’ large symptom improvements than the medium of treatment delivery.

## Highlights

• Sudden gains were common among participants in both CT (64%) and iCT (51%).
• Having a sudden gain was associated with better treatment outcomes.
• Changes in social cognition and self-focused attention occurred prior to sudden gains.
• Clients demonstrated generalised learning in sessions that preceded sudden gains.

## 1 Introduction

Sudden gains are large and stable decreases in clinical symptoms between consecutive therapy sessions (Tang & DeRubeis, 1999), which have been found in many studies of psychological therapies for mental health problems (Shalom & Aderka, 2020). A recent meta-analysis of 50 sudden gains studies found that 14.4–62.2% of treated patients experienced a sudden gain, and that sudden gains were associated with greater improvement on primary outcome measures at end of treatment and follow-up (Shalom & Aderka, 2020). Sudden gains therefore represent an interesting clinical phenomenon that may have implications for enhancing therapies if their occurrence can be better understood.

The criteria used to identify sudden gains specify that gains must be large in absolute magnitude, large in relative magnitude, and large relative to symptom fluctuation (Tang & DeRubeis, 1999; Tang, DeRubeis, Beberman, & Pham, 2005). There are currently five studies that have examined sudden gains in social anxiety disorder (SAD) (Bohn, Aderka, Schreiber, Stangier, & Hofmann, 2013; Butler et al., 2019; Hofmann, Schulz, Meuret, Moscovitch, & Suvak, 2006; Shalom et al., 2020; Thorisdottir, Tryggvadottir, Saevarsson, & Bjornsson, 2018). These have focused on CBT interventions in individual, group, and online formats, as well as interpersonal therapy and mindfulness-based cognitive therapy approaches. Results across the studies have been relatively consistent regarding the proportion of patients with SAD who experience a sudden gain. Typically around 20% of patients showed a sudden gain, a figure that is lower than the average proportion across all disorders studied (34.65%), as reported by Shalom and Aderka (2020). However, other findings in the SAD studies have been less consistent, particularly in two respects: firstly regarding whether experiencing a sudden gain is associated with better overall treatment outcomes, which is true of most sudden gains studies in other clinical conditions (Shalom & Aderka, 2020). Among the five SAD studies that assessed sudden gains, two (Bohn et al., 2013; Shalom et al., 2020) found that individuals who experienced sudden gains had better overall clinical outcomes and three (Butler et al., 2019; Hofmann et al., 2006; Thorisdottir et al., 2018) found no such relationship. Secondly, whether there is evidence of changes in anxious cognitions associated with the gain. Neither of the two studies that examined such cognitions (Bohn et al., 2013; Hofmann et al., 2006) found evidence of greater cognitive change in the session prior to a sudden gain. However, Bohn et al. (2013) found evidence of reduced negative social cognitions occurring concurrently with the sudden gain. Hofmann et al. (2006) found no evidence of concurrent cognitive change, but noted that the cognition measure used in their study, the Negative Self-Statements During Public Speaking scale (SSPS-N; Hofmann & DiBartolo, 2000) only assesses negative self-perception, which may not be the only cognitive change expected to occur across therapy. It is possible that the inconsistencies in findings across the SAD studies may stem from variability in methodology, for example, different treatment types, delivery formats, and process and outcome measures. There is therefore a need for further of sudden gains in the treatment of SAD before conclusions can be drawn.

A further question of interest in the sudden gains literature is which factors may predict their occurrence. This has been examined by analysing changes in cognitions or other theoretically-derived constructs prior to sudden gains (e.g. Wiedemann et al., 2020, Wiedemann et al., 2020), whether they can be predicted from demographic or clinical characteristics (e.g. Doane, Feeny, & Zoellner, 2010), or whether some sudden gains might be considered ‘false positives’ linked to measurement error, pretreatment variability or other statistical artefacts (see Shalom et al., 2020; Thomas & Persons, 2013; Vittengl, Clark, Thase, & Jarrett, 2015). Other studies have closely examined the content of the pre-gain session using therapy session videotapes to explore patient and therapist behaviours immediately preceding gains (e.g. Goodridge & Hardy, 2009; Lutz et al., 2013; Norton, Klenck, & Barrera, 2010; Tang & DeRubeis, 1999; Tang et al., 2005). This approach has the advantage of focusing specifically on within-session content, and by drawing on ratings from external observers, it reduces the need to rely exclusively on self-report data. However, this approach has yet to be taken within interventions for SAD.

This study aimed to examine the occurrence and possible determinants of sudden gains in individual cognitive therapy for SAD, comparing the standard face-to-face treatment format (CT) with a therapist-guided Internet-based programme (iCT). This is the first study of its kind to make a comparison of two delivery formats where the treatment content is identical. Sudden gains have been shown to occur in Internet interventions (e.g. Hedman et al., 2014; Shalom et al., 2020), but it is not yet known whether the frequency and nature of these gains are comparable to those occurring in the equivalent therapy delivered face-to-face. This is an important step to understand whether the psychological mechanisms of change underlying face-to-face and Internet-based treatments are similar. Lastly, we aimed to explore therapy content in more detail by examining videotapes of face-to-face CT sessions.

## 2 Method

### 2.1 Participants, treatments, and therapists

Data were drawn from a waitlist-controlled RCT (Clark et al., 2022) comparing face-to-face CT-SAD (n = 50) with therapist-guided internet-based iCT-SAD (n = 49). Participants all met DSM-IV (American Psychiatric Association, 1994) criteria for SAD, were age 18–65, were able to read English, had adequate access to an internet-enabled computer, and were on no or stable psychotropic medication. Exclusion criteria were current alcohol or substance dependency or borderline personality disorder, and current or past psychosis. The study was reviewed and approved by the local ethics committee.

Both treatments were delivered over 14 weeks with follow-up at three and twelve months. Three clinical psychologists with experience in both treatment formats delivered the treatments. The CT-SAD treatment protocol is based on the Clark and Wells (1995) model and is delivered via individual in-person sessions (Clark et al., 2006) on a weekly basis. Each session is 90 min duration and often includes the patient and therapist completing behavioural experiments together. The iCT-SAD intervention is a modular therapist-guided online treatment that delivers the same content and techniques as CT-SAD (see Stott et al., 2013). Therapist guidance is provided via a weekly phone call of approximately 20 min duration, plus other communication during the week through messaging and SMS. Treatment also includes a webcam experiment with the therapist in week two. The majority of behavioural experiments are done independently by the patient outside of the weekly calls.

### 2.2 Measures

Measures were completed prior to each weekly face-to-face CT session, and on a weekly basis for patients allocated to iCT. Although the RCT included a waitlist control group, outcome measurement during the wait period was insufficiently frequent (i.e. baseline, mid-wait, and post-wait) to allow the calculation of sudden gains in this condition. Sudden gains were calculated based on the *Liebowitz Social Anxiety Scale – Self-report version* (LSAS; Baker, Heinrichs, Kim, & Hofmann, 2002; Heimberg et al., 1999). Additional independent ratings of social anxiety were made using the relevant section of the *Anxiety Disorders Interview Schedule* (ADIS; Brown, Barlow, & DiNardo, 1994) at the start and end of treatment. The present study also examined changes in negative social cognitions and self-focused attention, which are central to the Clark and Wells (1995) model, around the period of the gain. There is evidence that these variables mediate treatment outcomes in CT-SAD (Hoffart, Borge, & Clark, 2016; Hoffart, Borge, Sexton, & Clark, 2009; Mörtberg, Hoffart, Boecking, & Clark, 2015; Thew et al., 2020). Depressed mood was also measured to examine the specificity of any effects observed for the model-derived variables. Negative social cognitions were assessed with the *Social Cognitions Questionnaire* (SCQ; Clark, 2005); self-focused attention (SFA) with two items of the *Social Phobia Weekly Summary Scale* (SPWSS; Clark, 2005); and depressive symptoms with the *Patient Health Questionnaire – 9-item version* (PHQ-9; Kroencke, Spitzer, & Williams, 2001).

### 2.3 Identification of sudden gains

Sudden gains were identified based on the LSAS, using the three criteria outlined by Tang et al. (2005). For criterion one, we calculated the reliable change index (RCI) as described in Jacobson and Truax (1991), using the standard deviation of the baseline assessment scores (SD = 18.0), and the internal consistency (Cronbach's alpha) of the scale (0.95; Baker et al., 2002), as shown below:

$$RCI=1.96\times \sqrt{{2\times \left(SD\times \sqrt{1-\alpha }\right)}^{2}}$$

Rounding up to the nearest whole number this gave a value of 12 points for the criterion one cutoff, which is in line with RCI calculations used in other studies of iCT-SAD (Stott et al., 2013; Thew et al., 2019) and is similar to the 10 point cutoff used in Hofmann et al. (2006). Gains were required to represent a drop of at least 25% of the pregain score (criterion two), and the mean difference between the three pregain and three postgain measurements was required to be greater than 2.78 times the pooled standard deviation of the two groups (criterion three). Where only five of the six datapoints were available (i.e., for gains between sessions 2 and 3, or between sessions 12 and 13, or where one datapoint was missing), an adjusted critical value of 3.18 was used (see Lutz et al., 2013). Gains occurring following the first session or the penultimate session were therefore not included.

The sessions before and after a sudden gain are referred to as session ‘n’ (pregain session) and session ‘n+1’ (postgain session), respectively, with earlier or later sessions identified in relation to session ‘n’ (e.g. session ‘n-2’). In iCT, where therapist contact is brief but regular throughout each week, and assessments are completed online on a weekly basis, the notion of therapy ‘sessions’ is not applicable; here, we use the term ‘week’, where ‘week x’ refers to the period between assessment x and assessment x+1.

### 2.4 Missing data

Where individual questionnaire item scores were missing, the total score was calculated by prorating missing scores based on the mean of the completed items. If an entire questionnaire was missed, the weekly total score was not estimated or replaced, to avoid influencing the calculation of sudden gains (see Tang, DeRubeis, Hollon, Amsterdam, & Shelton, 2007). Across the posttreatment, three-month follow-up, and 12-month follow-up assessments, five participants were missing at least one LSAS score, and 12 missing at least one ADIS score. When analysing the relationship of sudden gains to therapy outcomes, a linear mixed effects approach was used, which has the advantage of implicitly accounting for data missing at random.

### 2.5 Procedure for videotape ratings

In preparation for this study, the first author viewed a sample of recordings of six pregain (n) CT-SAD sessions along with their corresponding pre-pregain (n-1) sessions as a control. This led to a hypothesis that pregain sessions may be associated with a greater amount of generalised learning relative to control sessions. To test this, we developed an original coding manual to facilitate the rating of tapes blinded to whether they were a pregain or control session, and permit analysis of interrater reliability. The coding manual (see Supplementary Material) operationalised the concept of generalised learning i.e., new learning arising from any therapy activity that applies across a range of different social contexts and involves drawing a more general conclusion about the self, others, or social situations. Generalised learning (e.g., “*I am acceptable to others, and even when I feel anxious I come across well.*”) was contrasted with specific and/or situational learning, where a client's learning relates to a particular interaction or social situation (e.g. “*At the party, people were interested in what I was saying*.”). Four ratings were made for each session. The first two scales rated the extent to which the therapist asked questions aiming to elicit generalised learning (e.g. “*What does this say about the idea that you are unlikeable?*”) and specific learning (e.g. “*How did the other people respond on that occasion?*”), using a 0–2 scale (0 = no questions, 1 = a limited number of questions, 2 = numerous questions). The remaining two scales rated the extent to which clients' statements demonstrated the two forms of learning. These used 0–6 scales, with 0, 2, 4, and 6 indicating ‘no evidence’, ‘some but limited evidence’, ‘moderate evidence’, and ‘extensive evidence’ of that form of learning, respectively. A larger scale range was chosen given the more nuanced range of how client statements may indicate learning compared to the extent of therapists' questions.

To ensure blind ratings, the selection and ordering of therapy tapes to be rated was performed by an independent research assistant as follows. Firstly, the pregain and control sessions for each client experiencing a gain (n = 32) were identified. In five cases where participants had sudden gains in two consecutive sessions, the later session was not selected due to the absence of an appropriate control session, and in remaining cases where participants had more than one sudden gain, the largest gain was selected. Where a pregain session tape was not available (n = 7), this session and the corresponding control session were excluded, and where a control session tape was not available (n = 5), the previous session (n-2) was used as a replacement. This resulted in 25 tape pairs, each comprised of one pregain and one control session. An additional 13 pairs of sessions from participants who never experienced a sudden gain were added as foils (i.e., a 2:1 ratio). The session numbers of these foil pairs were selected to match the frequency of session numbers in the sudden gains group. Tape pairs were viewed together by the first author, with the order counterbalanced so that pregain tapes were viewed first 50% of the time. The order of tape pairs was randomised with respect to whether the client had experienced a sudden gain or not. To assess the reliability of the coding manual, a second rater (a clinical psychologist) independently scored a sample of eight tape pairs (21%). These ratings showed ICC(2,1) values between 0.76 and 0.85 indicating excellent agreement (Cicchetti, 1994) across the four rating scales.

### 2.6 Analysis

Analyses were performed in R version 3.4.3 (R Core Team, 2017) using the packages ‘suddengains’ (Wiedemann, Thew, Stott, & Ehlers, 2020), ‘nlme’ (Pinheiro, Bates, DebRoy, Sarkar, & R Core Team, 2018), and ‘tidyverse’ (Wickham, 2017), with additional analyses using SPSS version 24. Statistical assumptions were checked and met for each analysis.

## 3 Results

### 3.1 Frequency and Description of sudden gains

Of the 1089 between-session intervals analysed, 77 were classified as sudden gains (7%). In the CT condition, 32 participants (64%) experienced a sudden gain, 11 of whom experienced more than one, leading to a total of 44 sudden gains in this group. In the iCT condition, 25 participants (51%) experienced a sudden gain, with nine of those experiencing more than one, leading to 33 sudden gains in total for this group. Chi-squared tests showed that the proportion of patients experiencing a sudden gain did not significantly differ between treatment conditions (*χ*^2^ = 1.707, *p* = .191), nor the proportion of sudden gain sessions relative to the total number of inter-session intervals analysed (*χ*^2^ = 1.461, *p =* .227). Among patients who experienced a sudden gain, the proportion of patients with more than one sudden gain was not significantly different between treatment conditions (*χ*^2^ = 0.016, *p* = .898).

**Fig. 1:**
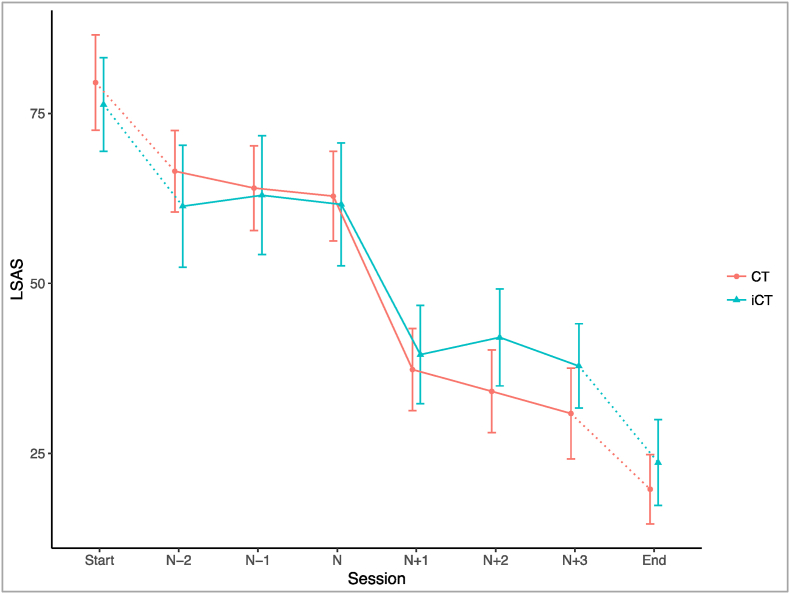
Mean scores on the Liebowitz Social Anxiety Scale (self-report version; LSAS) before and after sudden gains, defined as occurring between session/week n and session/week n+1, arranged by treatment condition. N = 57. Where a participant had more than one gain, their largest gain is included. Error bars represent 95%CI. CT = Cognitive Therapy; iCT = Internet-based Cognitive Therapy.

An independent samples *t*-test showed there was no significant difference in the mean magnitude of sudden gains in CT (M = 23.0, SD = 9.0) and iCT (M = 20.8, SD = 7.3): *t*(75) = 1.155, *p* = .252. On average, sudden gains represented 47% and 56% of participants’ overall improvement on the LSAS across the CT and iCT interventions, respectively,^1^ which is consistent with previous studies (e.g. 42.3% in Shalom & Aderka, 2020, p. 51% in Tang & DeRubeis, 1999). The distribution of the occurrence of sudden gains was relatively even across the course of treatment for both interventions, with the modal pregain sessions/weeks being five and twelve in CT and four in iCT. The average sudden gain for each treatment is shown in Fig. 1.

### 3.2 Relationship to therapy outcomes

**Fig. 2:**
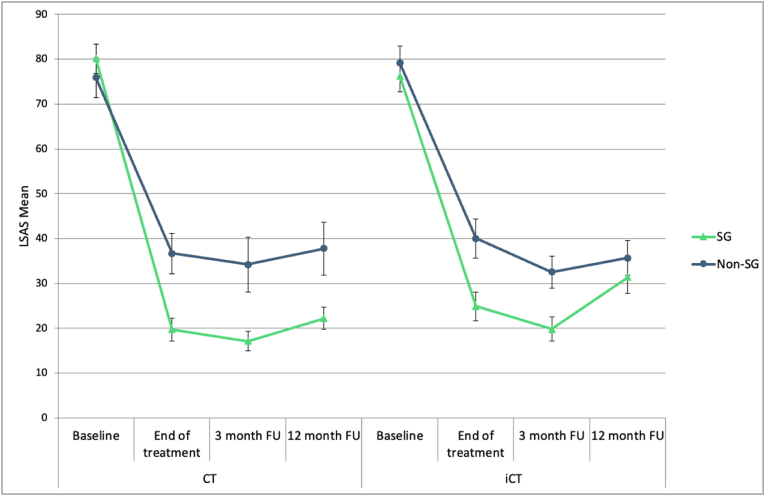
Mean scores on the Liebowitz Social Anxiety Scale (self-report version; LSAS) at Baseline, End of treatment, 3 and 12-month follow-ups, grouped by whether participants had a sudden gain during treatment (n = 57) or not (n = 42). Error bars represent ± 1SE. CT = Cognitive Therapy; iCT = Internet-based Cognitive Therapy; SG = Sudden Gain.

A linear mixed effects model was used to test for differences in therapy outcomes between participants who did and did not experience a sudden gain. This model used LSAS scores as the dependent variable, with categorical fixed factors of time (posttreatment assessment vs. three-month follow-up vs. 12-month follow-up), treatment condition (CT vs. iCT), and sudden gain status (present vs. absent). A random effect of participant was specified to account for between-subject variation. The baseline LSAS score was entered as a covariate. Models used maximum likelihood estimation, an unstructured covariance matrix, and retained cases with missing data. The assumption of normally distributed residuals was examined using Q-Q plots and found to be met. Pairwise comparisons indicated that participants with sudden gains showed lower LSAS scores than those without sudden gains at the posttreatment (adjusted difference = 16.40, SE = 3.41, *p* < .001, *d* = 0.91), 3-month follow-up (adjusted difference = 14.70, SE = 3.41, *p* < .001, *d* = 0.82), and 12-month follow-up (adjusted difference = 10.20, SE = 3.43, *p* = .004, *d* = 0.57) timepoints. Effect size estimates fell in the medium to large range, and most of the adjusted differences exceeded the 12-point Reliable Change Index of the LSAS, suggesting a clinically meaningful difference. As indicated in Fig. 2, baseline LSAS scores were not significantly different between those with and without gains, both in CT: *t*(48) = −0.775, *p* = .442, and iCT: *t*(47) = 0.568, *p* = .573 (unadjusted means and standard errors are shown in Table S1 of the supplementary material). The same pattern of results was found for the independent ratings of SAD symptoms using the ADIS, with pairwise comparisons indicating that participants with sudden gains showed lower ADIS scores than those without sudden gains at the posttreatment (adjusted difference = 0.77, SE = 0.19, *p* < .001, *d* = 0.78), 3-month follow-up (adjusted difference = 0.76, SE = 0.20, *p* < .001, *d* = 0.77), and 12-month follow-up (adjusted difference = 0.56, SE = 0.20, *p* = .005, *d* = 0.58) timepoints.

### 3.3 Changes on process measures around the gain

**Fig. 3:**
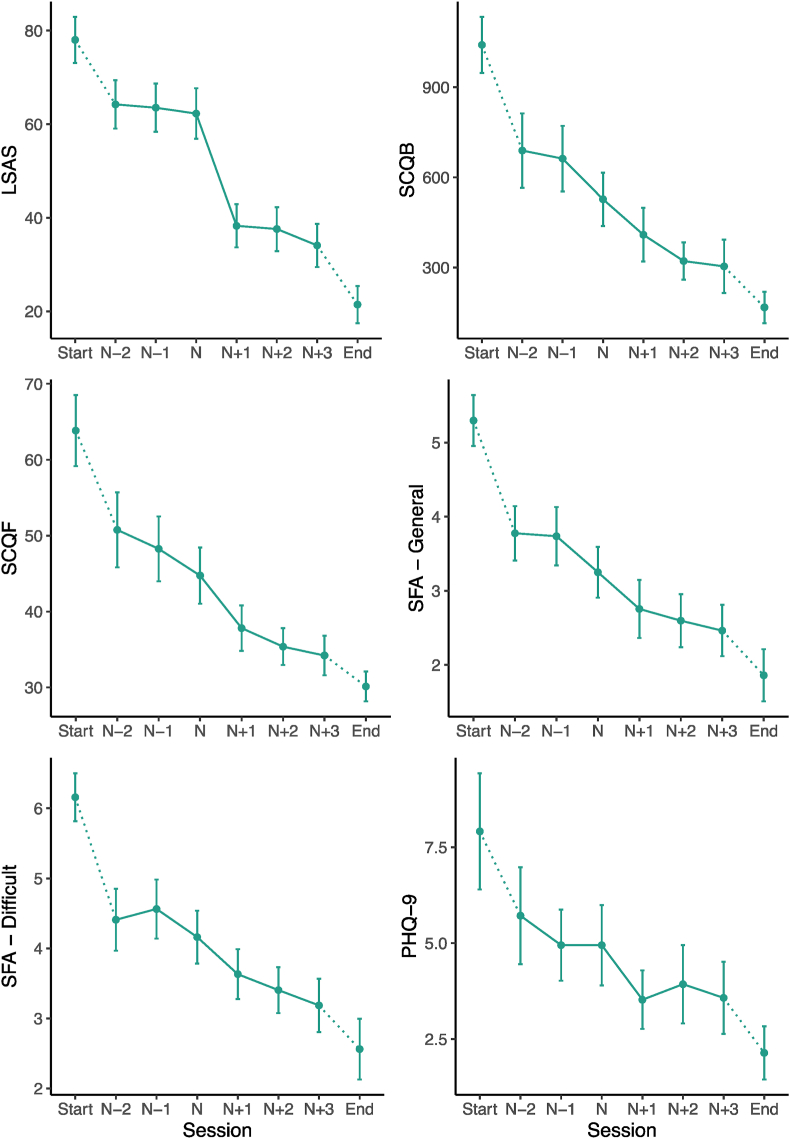
Mean scores on the LSAS and process measures around the gain. Sudden gains are calculated based on the LSAS, occurring between session n and session n+1. The two treatment conditions (CT and iCT) are collapsed as no group differences were shown. Error bars = 95%CI. N = 57. SCQF = SCQB = Social Cognitions Questionnaire – Belief; Social Cognitions Questionnaire – Frequency; SFA = self-focused attention (in difficult social situations, and in general); PHQ-9 = Patient Health Questionnaire (depression symptoms).

Participants’ scores around sudden gains on measures of negative social cognitions, self-focused attention, and depressed mood were analysed using linear mixed effects models, where timepoint (n-2, n-1, n, n+1, n+2, n+3) in relation to the gain was specified as a categorical fixed factor, and random intercepts specified to account for between-participant variation. Treatment condition was not specified as an additional fixed factor in order to simplify the models. Models were otherwise specified and assumptions examined as described earlier. Planned contrasts compared pairs of consecutive timepoints. Where participants had more than one sudden gain, their largest gain was used. Fig. 3 shows the mean scores on each measure around the sudden gain, and the results are given in Table 1.

**Table 1:** Results of linear mixed effects models examining process measures around sudden gains.

Contrast	*β* (SE)	*p*
SCQ-frequency		
n-2 to n-1	−3.21 (1.56)	.039
n-1 to n	−3.65 (1.50)	.015
n to n+1	−6.79 (1.50)	<.001
n+1 to n+2	−2.44 (1.49)	.101
n+2 to n+3	−1.46 (1.51)	.333
SCQ-belief		
n-2 to n-1	−52.52 (40.78)	.198
n-1 to n	−136.54 (39.16)	<.001
n to n+1	−116.73 (39.16)	.003
n+1 to n+2	−87.63 (38.95)	.024
n+2 to n+3	−20.19 (39.59)	.610
SFA general		
n-2 to n-1	−0.19 (0.20)	.352
n-1 to n	−0.50 (0.19)	.009
n to n+1	−0.48 (0.19)	.013
n+1 to n+2	−0.16 (0.19)	.410
n+2 to n+3	−0.16 (0.19)	.398
SFA difficult		
n-2 to n-1	0.03 (0.22)	.879
n-1 to n	−0.42 (0.21)	.042
n to n+1	−0.51 (0.21)	.016
n+1 to n+2	−0.23 (0.21)	.273
n+2 to n+3	−0.25 (0.21)	.232
PHQ-9		
n-2 to n-1	−0.85 (0.47)	.069
n-1 to n	−0.03 (0.45)	.949
n to n+1	−1.39 (0.45)	.002
n+1 to n+2	0.40 (0.44)	.364
n+2 to n+3	−0.41 (0.45)	.359

*Note*. N = 57 for all analyses. β = unstandardised mean difference. SCQ = Social Cognitions Questionnaire; SFA = self-focused attention; PHQ = Patient Health Questionnaire (depression symptoms).

Results indicated that negative social cognitions (SCQ-frequency and SCQ-belief), self-focused attention, and depressed mood (PHQ-9) showed a significant decline simultaneously with the sudden gain in social anxiety symptoms (n to n+1). Importantly, negative social cognitions and self-focused attention, but not depressive symptoms, also showed significant reductions immediately prior to the sudden gain (n-1 to n). For the SCQ-belief and self-focused attention scales, the results suggested this prior cognitive change was relatively circumscribed to the immediate pregain interval because the preceding interval (n-2 to n-1) did not show significant cognitive change. The SCQ-frequency subscale already showed a significant reduction over the n-2 to n-1 interval, which might indicate a cumulative effect, where cognitive changes build up over a few sessions leading to the sudden gain.

### 3.4 Rating of sudden gain videotapes in CT

**Fig. 4:**
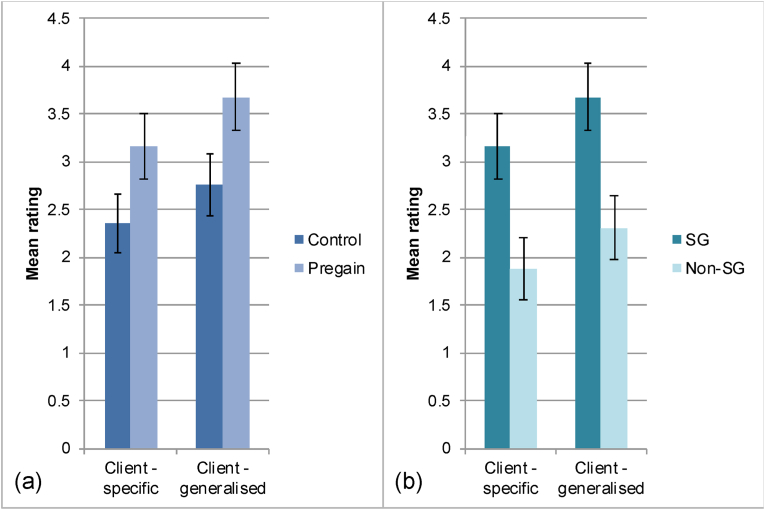
Results of the CT videotape ratings. Bars show the mean ratings of the extent to which client statements reflected specific and generalised learning. Panel a shows the within-participant analysis, comparing the pregain and control sessions of participants who had a sudden gain. Panel b shows the between-participant analysis, comparing the pregain sessions of those with a sudden gain (SG) and the sessions of participants not experiencing a sudden gain (Non-SG). Error bars represent ± 1SE.

Paired t-tests compared pregain and control sessions within participants who experienced a sudden gain. Results indicated that the ratings of clients' generalised learning were significantly higher for pregain sessions (M = 3.68, SD = 1.73) compared to control sessions (M = 2.76, SD = 1.59): *t*(24) = −2.554, *p* = .017, *d*_Cohen_ = 0.51. Ratings of participants' specific learning were not significantly different, *t*(24) = −1.979, *p* = .059. The equivalent comparisons for the therapist rating scales were nonsignificant: therapists' generalised learning questions, *t*(24) = 0.527, *p* = .603; therapists’ specific learning questions, *t*(24) = −0.296, *p* = .770 (see Fig. 4, panel a).

To evaluate the amount of learning between participants who did and did not experience a sudden gain, independent samples t-tests were used to compare the mean ratings for the pregain sessions of participants with a sudden gain, with the foil sessions of those without a sudden gain. Ratings of clients’ generalised learning, *t*(49) = −2.848, *p* = .006; and specific learning, *t*(49) = −2.694, *p* = .010; were significantly higher for participants with a sudden gain compared to participants without a sudden gain (see Fig. 4, panel b). No significant differences were observed between pregain and foil sessions in the extent to which therapists used generalised learning questions, *t*(49) = 0.178, *p* = .859; or specific learning questions, *t*(49) = −1.351, *p* = .183. Taken together, the videotape findings would suggest that generalised learning by the client is associated with the subsequent occurrence of a sudden gain, but the occurrence of generalised learning is not simply a function of therapists asking more generalised learning questions. Other aspects of the therapy process are presumably also contributing to generalised learning.

## 4 Discussion

### 4.1 Presence and frequency of sudden gains in CT and iCT

Sudden gains were found in both CT and iCT. The proportions of patients who showed sudden gains (64% and 51% respectively) were at the higher end of the range reported in a recent meta-analysis of all clinical conditions (Shalom & Aderka, 2020) and greater than the frequencies of around 20% reported in the other studies of sudden gains in SAD (Bohn et al., 2013; Butler et al., 2019; Hofmann et al., 2006; Shalom et al., 2020; Thorisdottir et al., 2018). The high frequency of sudden gains observed is perhaps not surprising given the high overall efficacy of cognitive therapy for SAD. In the trial from which the present data were drawn (Clark et al., 2022), the LSAS controlled pre-post effect sizes (*d*_*Cohen*_) against waitlist were greater than 2. In addition, nine previous randomized controlled trials in different countries have found CT-SAD to be superior to a range of alternative psychological and pharmacological interventions (Clark et al., 2003, 2006; Ingul, Aune, & Nordahl, 2014; Leichsenring et al., 2013; Mörtberg, Clark, Sundin, & ÅbergWistedt, 2007; Nordahl et al., 2016; Stangier, Heidenreich, Peitz, Lauterbach, & Clark, 2003; Stangier, Schramm, Heidenreich, Berger, & Clark, 2011; Yoshinaga et al., 2016). The protocol for CT-SAD that was used in both delivery formats contains a number of discrete techniques such as the self-focused attention and safety behaviours experiment, videofeedback, and techniques for addressing socially traumatic memories, that often generate key realisations for the client, and can lead to significant symptom reduction in their own right (McManus et al., 2009; Wild, Hackmann, & Clark, 2007). Besides the particular treatment used, it is conceivable that the high frequency of sudden gains in this study was partly due to small differences in the criteria for identifying sudden gains, though this seems unlikely to be a major contributor as the present study used a cutoff of 12 points on the LSAS for the first sudden gain criterion, which is marginally more conservative than the 10-point cutoff used in the only previous study using the LSAS to calculate gains (Hofmann et al., 2006).

The presence of sudden gains within iCT demonstrates these large symptom improvements can occur within online therapies, consistent with previous findings (e.g. Hedman et al., 2014; Shalom et al., 2020). This is the first study to compare sudden gains between face-to-face and internet-based treatments that are identical in content, and it is notable that in all analyses no evidence of significant differences between the two treatment formats were found. Online sudden gains were comparable in frequency, magnitude, and clinical significance to those in face-to-face CT treatment. This indicates that the therapy content and its implementation appears to play a more significant role in determining participants’ large symptom improvements, compared to the medium through which the therapy is delivered.

### 4.2 Relationship between sudden gains and treatment outcome

Consistent with the broader sudden gains literature, the presence of a sudden gain during therapy was associated with lower social anxiety scores at posttreatment, in both CT and iCT. Given that the baseline LSAS scores of participants who had a sudden gain did not differ from those without a sudden gain, the results support the view that sudden gains represent a meaningful and clinically significant shift, rather than simply artefacts of measurement. The association between having a sudden gain and improved outcome was maintained across the three-month and twelve-month follow-up assessments for both CT and iCT, consistent with Bohn et al. (2013) and Shalom et al. (2020).

### 4.3 Changes on process measures around the gain

Reductions in negative social cognitions and self-focused attention, two processes that are central to the Clark and Wells (1995) model, were observed prior to sudden gains in social anxiety, while changes in depressed mood were not. This suggests that cognitive and attentional changes during therapy may show a specific association with the presence of sudden gains, and may be processes that drive symptom improvement. This is the first study of sudden gains in SAD to find evidence of cognitive change occurring prior to gains. There was evidence to indicate that most of this change happens immediately prior to the gain, but findings from the SCQ-frequency subscale indicate that a cumulative effect from earlier sessions may also be possible, and this could usefully be explored further in future studies.

Sudden gains in social anxiety symptoms were associated with concurrent widespread improvements across other symptom and process variables. These include further improvements in cognitions and self-focused attention, and improvements in mood.

### 4.4 Videotape ratings in CT

The results from the videotape ratings suggested an association between generalised learning and the subsequent occurrence of a sudden gain. As generalised learning represents a form of cognitive shift, this is consistent with the earlier findings regarding prior cognitive change. While the amount of specific learning was greater in pregain sessions compared to control sessions, the difference was nonsignificant. One interpretation is therefore that the generalisation is particularly important, rather than the overall amount of learning. The dependent measure in this study (LSAS) assesses fear and avoidance across a wide range of social situations. Generalising learning from one situation to other situations would therefore be necessary to achieve a large improvement. As social anxiety generally starts early in life and natural recovery is rare (Bruce et al., 2005), generalisation may be particularly important in overcoming such chronic negative beliefs and symptoms. Work from educational psychology suggests that ‘knowledge transfer’ depends on noticing that the current situation is similar to one you have learned about previously, but that failing to recognise this is extremely common (Day & Goldstone, 2012). Gumport, Williams, and Harvey (2015) found that generalising, but not simply thinking about, nor applying the learning, was associated with improved outcomes one week later in CBT for depression. Generalised learning therefore appears to show quite specific effects and may represent a promising line of research with the potential to enhance treatment outcomes. It should be noted however, that the mean rating of generalised learning during pre-gain sessions fell just below ‘moderate’ on the scale. This suggests that not all participants showed substantial generalised learning in this session. This implies that other factors may also have played a role in the occurrence of sudden gains.

From a clinical perspective we may wish to consider whether and how therapists can facilitate more generalised learning, as it is possible that just one good question may make an important difference. Questions such as ‘What does this say about how acceptable you are to other people?’, ‘How do others respond to you generally, if you drop your safety behaviours and let them get to know you?’, or ‘Do you think this might apply in other situations?’ may facilitate clients considering this, potentially leading to greater therapeutic gains, and it would be important to examine this in future studies.

### 4.5 Limitations

First, as highlighted previously, although we followed standard criteria for identifying sudden gains, these are open to criticism. Concerns have been raised about the likelihood of ‘false positive’ sudden gains and thus their clinical significance, and although the present findings suggest this was unlikely, it remains possible that some of the gains found in this sample occurred by chance variation in scores. However, in the interest of consistency and comparability with other studies, we have retained the original criteria. Second, as the weekly questionnaire measures were completed immediately prior to each CT therapy session, it is not possible to distinguish gains that occurred within a session from those that occurred during the rest of the week leading up to the next session. Repeated measurement before and after face-to-face therapy sessions may help to explore within-session changes more directly, though may be impractical within some study designs. Third, there was no control group in this study that had the repeated measurement required to assess sudden gains. It is noted that only six participants in the waitlist condition (n = 34) showed LSAS improvements greater than 12 points in the seven weeks between baseline and mid-wait, and six participants in the seven weeks between mid-wait and post-wait. This suggests that sudden gains were perhaps less likely to have occurred in the waitlist group, but future studies could consider if weekly outcome measurement during a wait period is feasible, to permit the direct examination of sudden gains in the absence of treatment. Fourth, although the overall sample was relatively large, the subdivision between treatment conditions and sudden gain status resulted in more modest sample sizes per group, which may have limited statistical power. Replication in larger samples is warranted. Lastly, the coding manual used was developed specifically for this study, therefore has yet to be further validated in other studies.

### 4.6 Conclusions

This study has shown that sudden gains can occur in online therapies, and that these gains are similar in frequency, magnitude, and clinical significance to those seen in face-to-face CT. Sudden gains were common in both CT and iCT and were more frequent compared to previous studies of social anxiety treatments. Having a sudden gain predicted better overall therapy outcomes. The results provided evidence of reductions in negative social cognitions and self-focused attention occurring prior to sudden gains, but not changes in depressed mood. Clients’ statements in CT sessions indicated greater generalised learning in pregain compared to control sessions, suggesting a possible association between generalised learning and the occurrence of sudden gains. Taken together, the results support the view that sudden gains reflect clinically meaningful events in therapy, that the online therapy format does not diminish their likelihood, and that further investigation is warranted into how learning from therapy generalises and how this may influence outcomes.

## Funding

This work was supported by the 10.13039/100010269Wellcome Trust [102176 (GRT); 069777 and 200796 (AE & DMC)], the 10.13039/501100013373NIHR Oxford Biomedical Research Centre (GRT), and the Oxford Health NIHR Biomedical Research Centre (GRT, AE). The views expressed are those of the authors and not necessarily those of the NHS, the NIHR or the Department of Health.

## CRediT authorship contribution statement

**Graham R. Thew:** Conceptualization, Methodology, Formal analysis, Writing – original draft, Writing – review & editing. **Anke Ehlers:** Conceptualization, Methodology, Writing – review & editing, Supervision. **David M. Clark:** Conceptualization, Methodology, Writing – review & editing, Supervision.

## Declaration of competing interest

None.

## Data Availability

Data in relation to this study will be made available upon reasonable request.

## Footnotes

1 These percentages include only the largest gain where a participant had more than one.
