# Supplementary material for: Sudden gains in face-to-face and internet-based cognitive therapy for social anxiety disorder
Source: Behav Res Ther. 2023 Jul;166:104334. doi: 10.1016/j.brat.2023.104334 (PMC10933765; doi:10.1016/j.brat.2023.104334)
Supplement: Multimedia component 1 [file mmc1.pdf]

Supplementary Material to:

Thew, G.R., Ehlers, A., & Clark, D.M. Sudden gains in face-to-face and internet-based Cognitive Therapy for Social Anxiety Disorder

Table S1

Unadjusted means and standard errors of LSAS scores at each timepoint.

		LSAS Mean (SE) [N]	
		Sudden Gain	No Sudden Gain
CT	Pre	80.1 (3.3) [32]	75.9 (4.4) [18]
	Post	19.7 (2.6) [32]	36.7 (4.5) [18]
	3 months	17.1 (2.2) [31]	34.2 (6.1) [18]
	12 months	22.2 (2.5) [31]	37.8 (5.9) [17]
iCT	Pre	76.3 (3.5) [25]	79.2 (3.7) [24]
	Post	24.9 (3.2) [25]	40.0 (4.3) [23]
	3 months	19.8 (2.7) [24]	32.5 (3.5) [24]
	12 months	31.4 (3.7) [25]	35.7 (3.9) [23]

Notes. LSAS = Liebowitz Social Anxiety Scale; SD = Standard Deviation; CT = Cognitive Therapy; iCT = Internet-delivered Cognitive Therapy.

Coding Manual

Cognitive Therapy for Social Anxiety Disorder: Generalisation of Learning

Clients undertaking CT for SAD complete a number of behavioural experiments and other therapeutic activities that aim to facilitate new learning about themselves and how they come across in social situations.

There are two categories of learning which may distinguish the extent to which people respond to treatment sessions. The first concerns learning that is confined to specific situations discussed or experienced in therapy sessions. The second concerns learning that is more generalised. These categories are defined below.

1. Specific and/or situational learning vs. Generalised learning

1.1 General description of concept

The degree to which learning is generalised is thought to be a continuum, with the above two opposing constructs at each end. By definition, generalised learning must be generalised from something specific, so cannot be present in isolation. In contrast, specific and/or situational learning can occur without the presence of generalised learning. The constructs are defined as follows:

Specific and/or situational learning refers to learning that arises from any therapy activity (for example, discussion of homework, deriving a model, behavioural experiments, surveys, videofeedback, reviewing beliefs/rules, memory rescripting, attention training, addressing anticipatory worry or post-event rumination) that is limited to a particular event i.e., a certain conversation or interaction. This learning can be about the self (e.g., 'I didn't look as anxious as I felt in that conversation'), how others respond (e.g., 'that person seemed interested in what I was saying'), and/or about the social situation (e.g., 'The conversation was easier when I was externally focused'). However, in any of these instances the learning is restricted to drawing conclusions about what happened in that event.

Generalised learning refers to learning that arises from any therapy activity (for example, discussion of homework, deriving a model, behavioural experiments, surveys, videofeedback, reviewing beliefs/rules, memory rescripting, attention training, addressing anticipatory worry or post-event rumination) that applies across a range of different social contexts and involves drawing a more general conclusion about the self (e.g., 'I am acceptable'), others (e.g., 'Other people are interested in what I have to say'), or social situations (e.g., 'Conversations go better when I'm out of my head'). Generalised learning is often an extension of specific learning; an individual may demonstrate that they have learned a point in relation to a specific situation, but also that this learning will generalise to a range of other situations.

Rating the extent to which these forms of learning occur within a therapy session requires the consideration of two components: 1) the degree to which the therapist asks questions in order to elicit these forms of learning; 2) the degree to which statements made by the client reflect these forms of learning.

1.2 Evidence for Specific and/or situational learning

1.2.1 Therapist behaviours

Evidence that the therapist is attempting to elicit specific and/or situational learning will be taken primarily from the questions they ask, but may also be taken from other

behaviours such as using a behavioural experiment record sheet, where learning is recorded.

Questions:

At any point within a session, the therapist may ask questions related to a completed component of therapy (for example a piece of completed homework, a conversation, or behavioural experiment). These questions will relate specifically to the completed activity, and most often are quite specific and closed.

E.g. *How much did that person [the client] look shaky on the video?*
E.g*. How much did you sweat, 0-100?*
E.g. *Were your predictions correct?*
E.g. *How did the other people respond?*
E.g. *What was the difference between your prediction and what actually happened?*
E.g. *What effect did dropping your safety behaviours have on the conversation?*
E.g. *Did that person respond in a way that suggested you were unlikeable?*

Do not count general questions about how they found/experienced the activity.
Do not count questions about extending/repeating the activity.

1.2.2 Client statements

Evidence of specific/situational learning on the part of the client will be drawn from the statements they make at any point in a therapy session. They may be prompted by a question from the therapist or may be given spontaneously.

Statements in direct relation to the activity

E.g. *I don't/didn't look as red as I thought.*
E.g. *No-one was staring even though it felt like it.*
E.g. *When I focused more on the conversation it flowed better.*
E.g. *People didn't notice/weren't bothered when I said something boring.*
E.g. *People were interested in what I was saying.*

1.3 Evidence for Generalised learning

1.3.1 Therapist behaviours

Evidence that the therapist is attempting to elicit generalised learning will be taken primarily from the questions they ask, but may also be taken from other behaviours such as using a behavioural experiment record sheet, where learning is recorded.

Questions:

The therapist may ask questions related to the previous therapy activity (for example a piece of completed homework, a conversation, or behavioural experiment). These questions will make reference to the completed activity, but will aim to elicit broader learning. Both open and closed questions may be used.

E.g. *Do you think this might be true in other social situations?*
E.g. *What does this say about you more generally?*
E.g. *What does this tell you about how you come across to other people?*
E.g. *What does this say about how reliable our feelings are as a way to judge how we come across?*
E.g. *What does this say about the idea that you are unlikeable?*
E.g. *What effect do these safety behaviours have on how self-conscious you feel?*

<u>Do not</u> count questions about extending/repeating the activity.

1.3.2 Client statements

Evidence of generalised learning on the part of the client will be drawn from the statements they make at any point during a session. They may be prompted by a question from the therapist or may be given spontaneously.

<u>Statements about the self:</u>
E.g. *Even when I feel anxious I come across well.*
E.g. *I am able to speak to people I don't know very well.*
E.g. *I am acceptable to others.*

<u>Statements about others and social situations:</u>
E.g. *Conversations go better when I'm out of my head.*
E.g. *Other people are interested in what I have to say.*
E.g. *I don't have to be witty/intelligent in every conversation.*
E.g. *If I stumble over my words at work, people probably won't notice/care.*
E.g. *People have accepted me much more than I realised.*
E.g. *My feelings are private, and not visible to others.*
E.g. *Focusing on myself makes the negative thoughts seem worse.*

Evidence against generalised learning would include statements that indicate doubt about whether the learning would generalise:

E.g. *I think that person was just being nice.*
E.g. *I'm not sure people would be like that where I work.*
E.g. *I think I'd need to try it out again somewhere else.*
E.g. *That safety behaviour is probably unhelpful.*

<u>Do not</u> count generalised statements that are accompanied by an expression of doubt (e.g. *People are happy to help when I ask a question…though that might be different in London*)

Where a generalised statement is proposed or 'offered' by the therapist, clear evidence of the client's agreement with this is required to be considered evidence for generalised learning. Raters should consider whether the client's response to the statement indicates they are convinced and in agreement.

1.4 Procedure

YOUR TASK AS A RATER IS TO IDENTIFY SESSION CONTENT THAT COULD REPRESENT EVIDENCE FOR SPECIFIC AND/OR SITUATIONAL LEARNING AND GENERALISED LEARNING ON THE PART OF THE CLIENT, AND THERAPIST BEHAVIOURS DESIGNED TO ELICIT THIS.

1. When viewing the session tape, summarise the evidence for specific and/or situational learning AND generalised learning separately based on the instructions given above. This summary should distinguish between therapist behaviours, and client statements.

2. After viewing the entire session review the evidence you have collated, and then with reference to the scales below, give four overall ratings as described below.

3. Rate the extent to which therapist behaviours eliciting specific and/or situational learning are present in the session. Ratings of therapist behaviours should be made independently of how the client responds i.e., the therapist's question should be considered regardless of whether or how it is responded to.

   Rating:

   0 Therapist does not ask questions to elicit specific/situational learning from the activities completed in the session

1 Therapist asks a limited number of questions to elicit specific/situational learning from the activities completed in the session

2 Therapist asks numerous questions to elicit specific/situational learning from the activities completed in the session

4. Rate the extent to which therapist behaviours eliciting generalised learning are present in the session.

Rating:

0 Therapist does not ask questions to elicit generalised learning from the activities completed in the session

1 Therapist asks a limited number of questions to elicit generalised learning from the activities completed in the session

2 Therapist asks numerous questions to elicit generalised learning from the activities completed in the session

5. Rate the extent to which the client demonstrates specific and/or situational learning in the session.

Rating:

0 No evidence of specific and/or situational learning

1

2 Some, but limited evidence of specific and/or situational learning

3

4 Moderate evidence of specific and/or situational learning

5

6 Extensive evidence of specific and/or situational learning

6. Rate the extent to which the client demonstrates generalised learning in the session.

Rating:

0 No evidence of generalised learning

1

2 Some, but limited evidence of generalised learning

3

4 Moderate evidence of generalised learning

5

6 Extensive evidence of generalised learning

NOTE: The four rating scales are independent, and not mutually exclusive. For example, a session may have high scores for both client specific and/or situational learning and client generalised learning, high scores for one but not the other, or low scores for both. Equally, the therapist may ask few questions designed to elicit learning in a session thus receiving low scores, but the client's statements in that session may receive high scores on one or both scales if the relevant learning is indicated.
